# Pancreatic enzymatic mediastinitis followed by total gastrectomy with splenectomy: report of two cases

**DOI:** 10.1186/s40792-021-01240-5

**Published:** 2021-06-27

**Authors:** Yoko Zaitsu, Takashi Nishizaki, Takuma Izumi, Daisuke Taniguchi, Yuichiro Kajiwara, Yumi Oshiro, Kazuhito Minami

**Affiliations:** 1 Department of Gastroenterological Surgery, The Cancer Institute Hospital Of Japanese Foundation for Cancer Research, 3-8-31 Ariake, Koto-ku, Tokyo, 135-8550 Japan; 2 Department of Surgery, Matsuyama Red Cross Hospital, 1 Bunkyo-Cho, Matsuyama, Ehime 790-8524 Japan; 3 Department of Diagnostic Pathology, Matsuyama Red Cross Hospital, 1 Bunkyo-Cho, Matsuyama, Ehime 790-8524 Japan

**Keywords:** Pancreatic enzymatic mediastinitis, Total gastrectomy, Splenectomy, Pancreatic fistula, Pancreatic necrosis

## Abstract

**Background:**

Acute mediastinitis is a rare disease that rapidly progresses with a high mortality rate. Its most common cause is direct injury of the mediastinum, including iatrogenic causes such as cardiac surgery or upper endoscopy. Enzymatic mediastinitis is a rare complication of a pancreatic fistula caused by the inflammatory digestion of the parietal peritoneum spreading to the mediastinum. Here, we present two cases of enzymatic mediastinitis caused by total gastrectomy with splenectomy. One of them was successfully treated and cured after early diagnosis and transabdominal drainage.

**Case presentation:**

Case 1 was that of a 60-year-old man (body mass index [BMI] 27) with a medical history of diabetes and hypertension who was diagnosed with advanced gastric cancer in the upper body of the stomach. A total gastrectomy with splenectomy was performed. The patient experienced acute respiratory failure 24 h after surgery. Pulmonary embolism was suspected, so a computed tomography (CT) scan was performed; however, no relevant causes were found. Although he was immediately intubated and treated with catecholamine, he died in the intensive care unit (ICU) 40 h after surgery. Post-mortem findings revealed retroperitonitis caused by a pancreatic fistula spreading towards the mediastinum, causing severe mediastinitis; a review of the CT scan revealed pneumomediastinum. We concluded that the cause of death was enzymatic mediastinitis due to post-gastrectomy pancreatic fistula. Case 2 involved a 61-year-old man (BMI 25) with a medical history of appendicitis who was diagnosed with advanced gastric cancer at the gastric angle between the lesser curvature and the pylorus, spreading to the upper body of the stomach. A total gastrectomy with splenectomy was also performed. The patient had a high fever 3 days after the surgery, and a CT scan revealed pneumomediastinum, indicating mediastinitis. As the inflammation was below the bronchial bifurcation, we chose a transabdominal approach for drainage. The patient was successfully treated and discharged.

**Conclusion:**

Acute mediastinitis caused by gastrectomy is rare. The acknowledgment of abdominal surgery as a cause of mediastinitis is important. In treating mediastinitis caused by abdominal surgery, transabdominal drainage may be a minimally invasive yet effective method if the inflammation is mainly located below the bifurcation of the trachea.

## Background

**Fig. 1:**
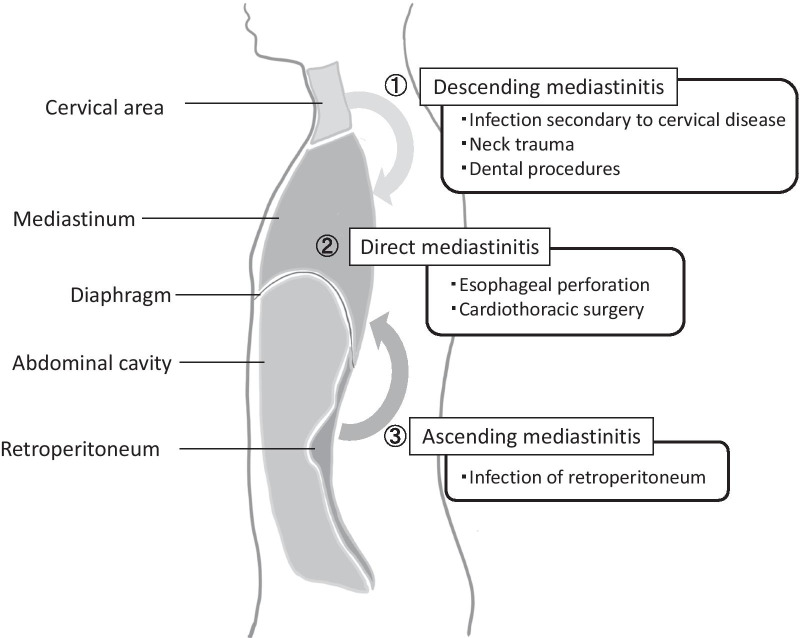
Causes of mediastinitis. Mediastinitis can be divided into three types, descending, direct, and ascending mediastinitis, according to the location of the infection and the way the infection spread

Acute mediastinitis is a rare post-operative complication. Its incidence in cardiac surgery ranges from 1 to 2% with a 14% to 47% mortality rate [1]. As the mediastinum is a sterile space, the infection occurs either from direct injury (i.e., direct mediastinitis) or an infectious extension (e.g., ascending mediastinitis, descending mediastinitis) (Fig. 1). Among these, direct mediastinitis is the most common type of mediastinitis. This is followed by descending mediastinitis, which is known to occur due to cricopharyngeal infection; gravity, breathing, and negative intrathoracic pressure facilitate the spread of the infection [2]. As the diaphragm separates the abdominal cavity and mediastinum, ascending mediastinitis caused by abdominal surgery is very rare. Among the few reports of mediastinitis induced by gastrectomy, rupture of esophagoenterostomy that directly affected the mediastinum was the main cause [3–5]. Abdominal infection rarely extends to the mediastinum; although it is rare, enzymatic mediastinitis is a type of acute mediastinitis caused by a pancreatic fistula [6, 7]. To better illustrate this, we report two cases of enzymatic mediastinitis due to total gastrectomy. In both cases, the esophagoenterostomy was intact, and severe retroperitonitis was observed around the pancreatic tail extending to the mediastinum, suggesting the presence of severe retroperitonitis caused by a pancreatic fistula.

## Case presentation

### Case 1

**Fig. 2:**
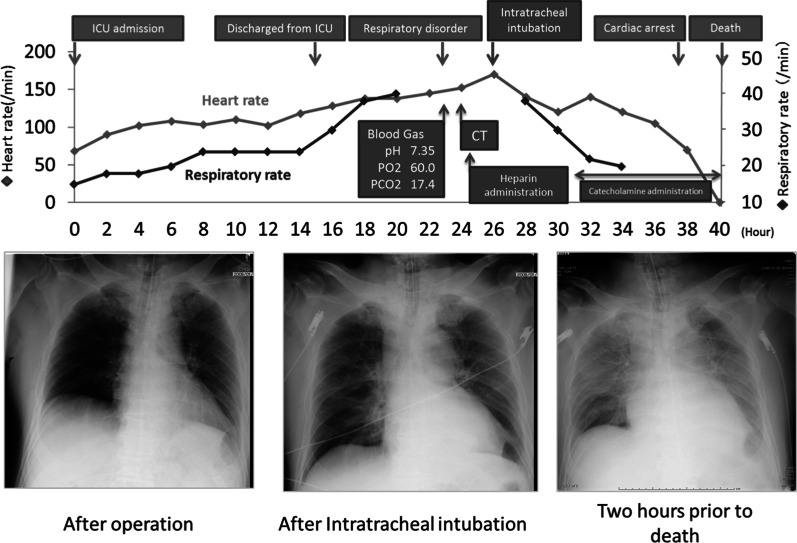
Postoperative course. The respiratory failure progressed rapidly, and the patient was diseased 14 h after intratracheal intubation

**Fig. 3:**
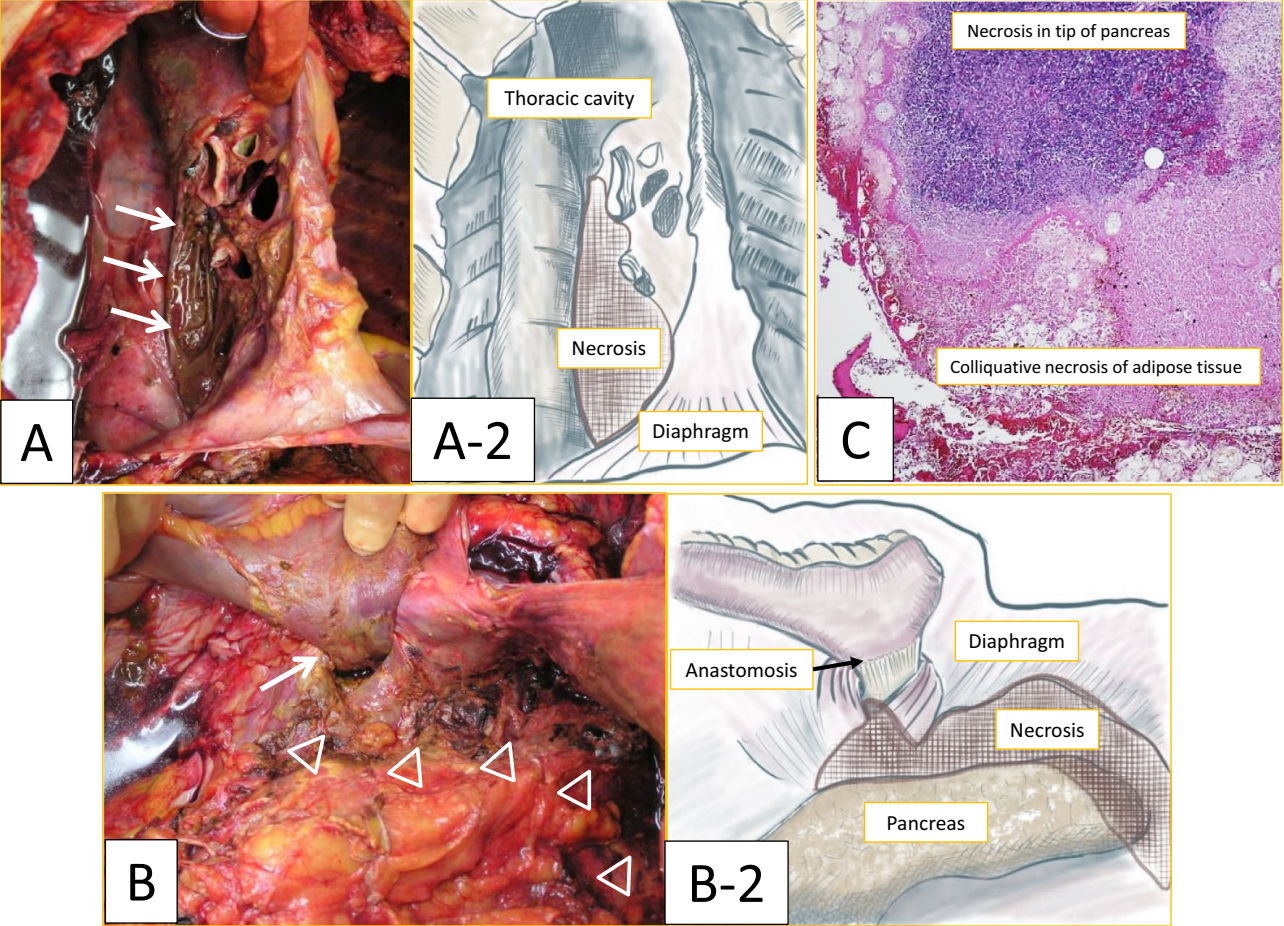
Post-mortem findings. **A** Macroscopic finding of the right side of the mediastinum, necrosis is observed in the mediastinum. **A-2** A schema of figure A. **B** Macroscopic finding of the posterior side of the anastomosis (arrow), the anastomotic site is intact, and continuous necrosis is observed around the pancreatic tail (△) to the mediastinum. **B-2** A schema of figure B. **C** Microscopically the surrounding adipose tissue of the pancreas showed colliquative necrosis. The inflammation was especially severe in the tail of the pancreas causing necrosis in the tip of the pancreatic tail

**Fig. 4:**
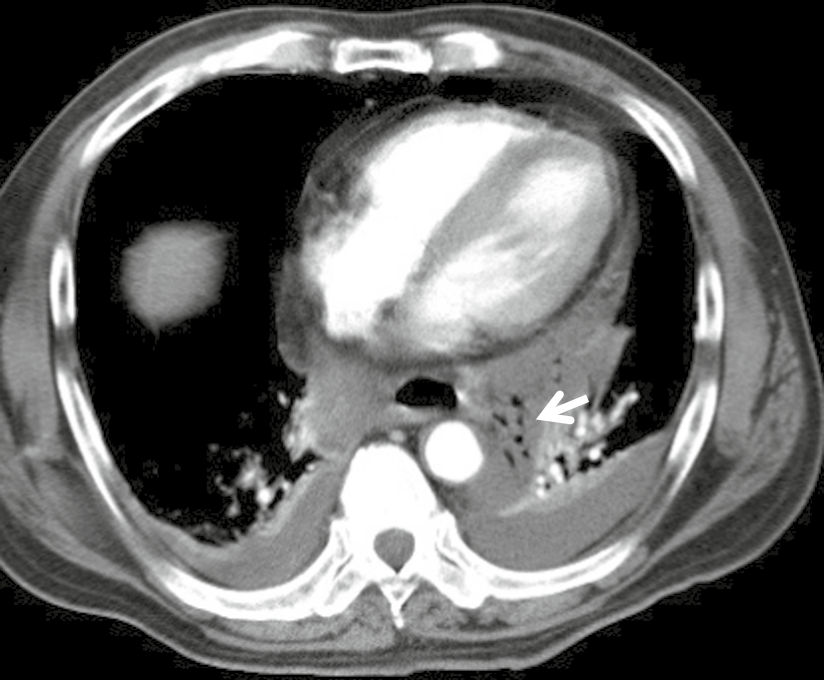
CT findings. Pneumomediastinum is shown indicating mediastinitis (arrow)

A 60-year-old Japanese man (body mass index [BMI] 27) with a medical history of diabetes and hypertension was diagnosed with advanced gastric cancer in the upper body of the stomach. Total gastrectomy with splenectomy and D2 lymph node dissection were performed as indicated by the Japanese gastric cancer treatment guidelines before 2018. The operation time of the patient was prolonged due to the thick visceral fat and firm adhesions observed on the lateral side of his spleen. The splenic artery was ligated and cut near the splenic hilum to preserve blood flow to the pancreas. The surgical time elapsed was 6 h and 45 min, and the total blood loss was approximately 1,499 mL. Two drains were placed on the right side of the esophagoenterostomy and below the left diaphragm. The final pathological finding was pT2N0M0, pStage IB (Japanese Classification of Gastric Carcinoma, 15th edition). The post-operative course is shown in Fig. 2. The patient experienced acute respiratory failure 24 h after surgery. Additionally, the drain inserted behind the esophagoenterostomy was intact, but the drain placed below the left diaphragm showed a dark red color. His drain amylase level was 16,800 U/L. Suspecting a pulmonary embolism, we performed a computed tomography (CT) scan, but no relevant causes were found. Although the patient was immediately intubated and treated with catecholamine, he died of cardiac arrest in the intensive care unit (ICU) 40 h after surgery. Post-mortem findings revealed vast necrosis affecting the entire mediastinum and accompanied by abscess formation, indicating severe mediastinitis (Fig. 3A, arrow). The site of anastomosis was intact (Fig. 3B, arrow), but continuous inflammation was observed from the tail of the pancreas to the mediastinum (Fig. 3B, arrowhead). The abscess from the mediastinum was positive for Gram-negative bacilli, and the surrounding adipose tissue of the pancreas showed colliquative necrosis, suggesting the formation of a pancreatic fistula. The inflammation caused by the pancreatic fistula was especially severe in the tail of the pancreas, causing necrosis at the tip of the pancreatic tail (Fig. 3C). Based on these findings, we concluded that a pancreatic fistula due to gastrectomy caused enzymatic mediastinitis. A review of the CT scan taken during the respiratory disorder revealed pneumomediastinum (Fig. 4, arrow). Post-gastrectomy of the patient, acute mediastinitis caused by anastomosis rupture or abdominal infection should have been included in his differential diagnosis after seeing air in the mediastinum.

### Case 2

**Fig. 5:**
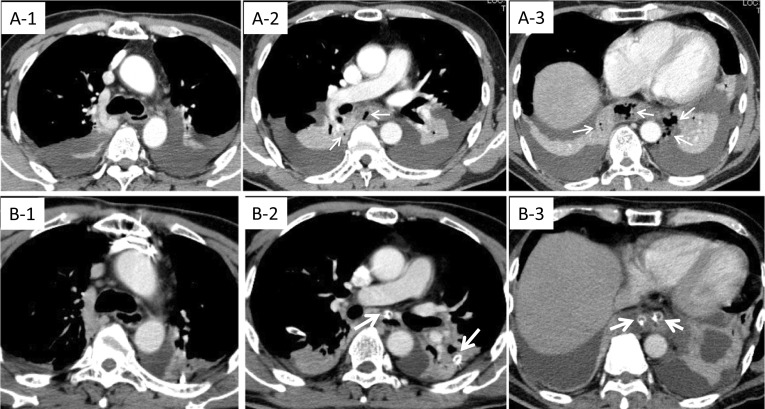
CT findings before (**A-1,2,3**) and after (**B-1,2,3**) an emergency operation. **A1 -3.** CT before emergency surgery. **A-1** Pneumomediastinum is not seen in the branchial bifurcation. **A-2,3** Pneumomediastinum is observed (arrow). **B1-3** CT after 4 days from emergency surgery**. B-1** Pneumomediastinum did not spread above the branchial bifurcation. **B-2,3** The drain placed from the abdomen is effective in reducing pneumomediastinum and inflammation

**Fig. 6:**
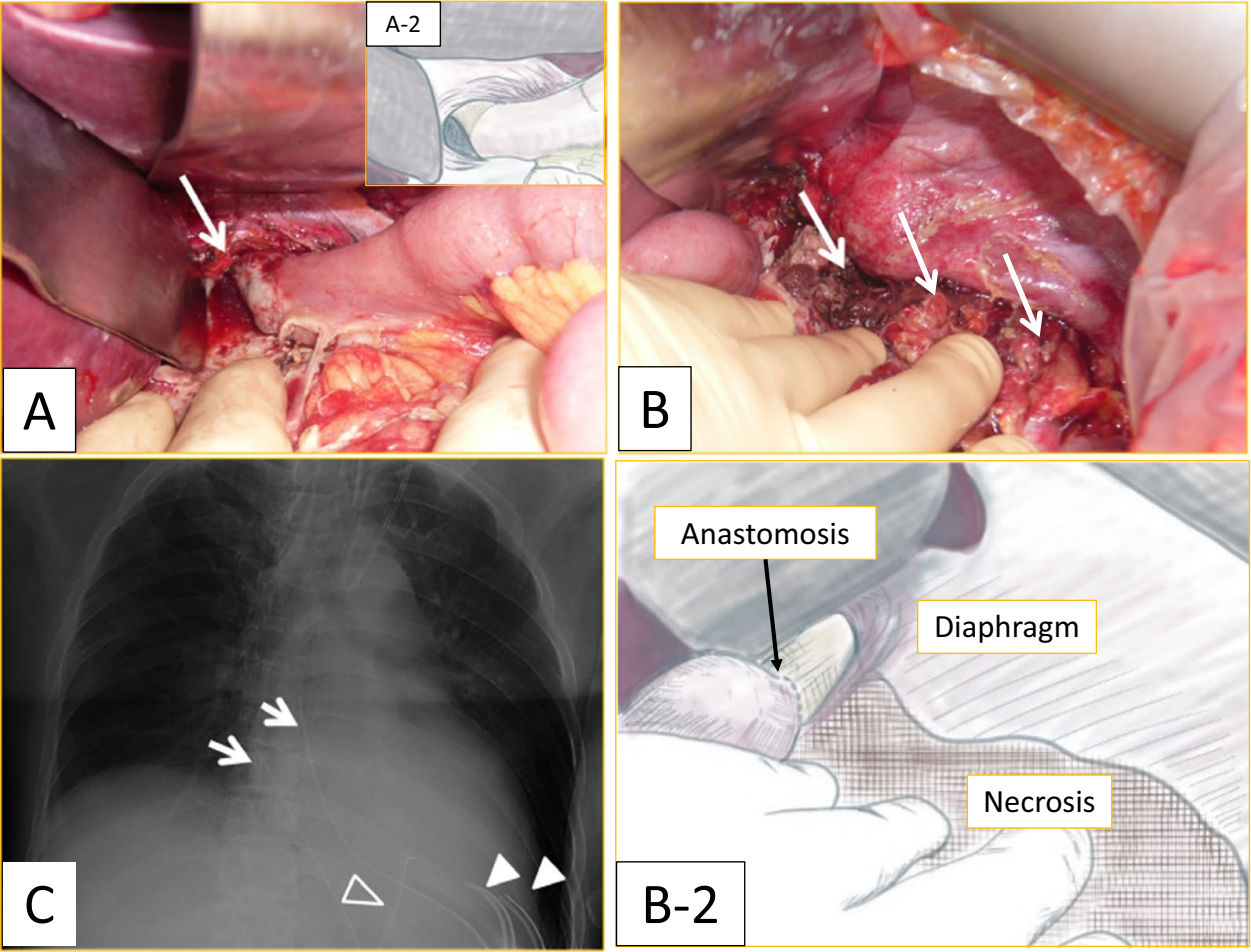
Operative findings. **A** The anastomosis (arrow) is intact. **A-2** A schema of figure A. **B** Continuous inflammation and necrotic tissue are observed from the pancreatic tail to the anastomosis site (arrow). **B-2** A schema of figure B. **C** Drain in the mediastinum (arrow), subphrenic area (▲), and the anastomosis site (△)

A 61-year-old man (BMI 25) with a medical history of appendicitis was diagnosed with advanced gastric cancer at the gastric angle between the lesser curvature and the pylorus with a poorly differentiated component spreading to the upper body of the stomach. Total gastrectomy with splenectomy and D2 lymph node dissection were performed as indicated by the Japanese gastric cancer treatment guidelines before 2018. The splenic artery was ligated and cut near the splenic hilum to preserve blood flow to the pancreas. Two drains were placed, one below the esophagoenterostomy and another below the left diaphragm. The total surgical time elapsed was 5 h and 56 min, and the total blood loss was approximately 167 mL. The final pathological finding was pT4aN3aM0, Stage IIIB (Japanese Classification of Gastric Carcinoma, 15th edition). Both drains turned dark red one day after surgery, and the drain amylase levels were measured at 2,904 U/L and 5,203 U/L below the esophagoenterostomy and below the left diaphragm, respectively. Although the drain discharge cleared and the drain amylase level decreased to 575 U/L, the patient developed a high fever 3 days after surgery. A CT scan revealed fluid around the retroperitoneum and near the pancreas. This continued to the mediastinum and was accompanied by pneumomediastinum below the branchial bifurcation (Fig. 5, A-1,2,3). As the patient showed signs of a pancreatic fistula, the possibility of enzymic mediastinitis was considered. Emergency drainage surgery was therefore immediately performed. As the inflammation was found below the bronchial bifurcation (Fig. 5, A-1), we chose a transabdominal approach for drainage. Although there were no ruptures at the site of anastomosis (Fig. 6A, arrow), there was continuous inflammation and abscess formation from the pancreatic tail to the mediastinum (Fig. 6B, arrow), indicating enzymatic mediastinitis. Five drains were inserted, two to the mediastinum (Fig. 6C, arrow), two to the subphrenic area (Fig. 6C, white arrowhead), and one near the esophagoenterostomy (Fig. 6C, arrowhead). Efficient drainage was obtained (Fig. 5, B-1,2,3), and the patient was successfully treated and discharged 124 days after the emergency surgery.

## Discussion

Mediastinitis is mainly caused by direct infection of the mediastinum. This is called direct mediastinitis, and cardiovascular surgery is the most common cause whereas 90% of nonsurgical mediastinitis occurs from esophageal perforations [8]. As the mediastinum is surrounded by loose connective tissue, mediastinitis can be caused by infection of the odontogenic, pharyngeal, or cervical areas [9], which is called descending mediastinitis. On the other hand, mediastinitis caused by infection from the retroperitoneum is called ascending mediastinitis. Considering these, enzymatic mediastinitis is classified as ascending mediastinitis. More specifically, digestion of the parietal peritoneum by pancreatic enzymes and the passage of inflammation to the thoracic cage through the esophageal or aortic hiatus has been suggested as its possible mechanism [6, 7]. This is generally associated with acute pancreatitis [7].

Mediastinitis caused by total gastrectomy is very uncommon, and among the few reports, all were caused by esophagojejunal anastomosis leakage [3–5]. The documented cases are presented in Table 1.

**Table 1:** Documented cases of mediastinitis caused by total gastrectomy

No.	Cause	Treatment	Result	References
1	Anastomotic leakage	Thoracic tube insertion	Alive	[3]
2	Anastomotic leakage	Total esophagectomy	Death	[4]
3	Anastomotic leakage	Total esophagectomy	Death	[4]
4	Anastomotic leakage	Total esophagectomy	Death	[4]
5	Anastomotic leakage	Total esophagectomy	Alive	[4]
6	Anastomotic leakage	Total esophagectomy	Death	[5]
7	Pancreatic fistula	Symptomatic treatment	Death	Present case 1
8	Pancreatic fistula	Surgical drainage	Alive	Present case 2

In document retrieval, a combination of keywords such as “Mediastinitis”, “Total gastrectomy”, “Pancreatic fistula”, “Anastomotic leakage” was used. Mediastinitis due to relapse or perforation of the tumor before surgery is not included. Note that reference No. 23 was a review of 198 patients that underwent surgery for type I and II esophagogastric junction adenocarcinoma

Pancreatic fistula is a well-known complication of gastrectomy. Damage to the pancreatic parenchyma and blood flow disorder due to lymph node dissection of the upper edge of the pancreas are known to be the causes of this complication [10, 11], and the incidence increases when splenectomy is performed [12, 13]. Infection or inflammation caused by a pancreatic fistula rarely affects the mediastinum as the thoracic cavity is separated from the abdominal cavity, and gravity prevents infection or inflammation from spreading to the mediastinum. In our two cases, transabdominal thoracotomy was not performed, but the peritoneum was cut open for total gastrectomy; therefore, continuity of the mediastinum and abdominal cavity was formed. As the patient must be in a supine position after surgery, the negative pressure of the mediastinum might have facilitated the pancreatic enzyme to spread toward the mediastinum. Mediastinitis associated with Gram-negative bacilli is known to be more severe than that caused by Gram-positive bacilli [14]. Bacteria such as *Pseudomonas aeruginosa* and *Enterobacter cloacae*, which are commonly seen in the intestines, are known to activate trypsinogen [15]. A randomized trial in patients with severe acute pancreatitis showed that mortality increases dramatically when Gram-negative infection of pancreatic necrosis occurs, and the source of infection is usually translocated from the intestines [16]. Although it is rare, total gastrectomy might have a higher incidence of developing an enzymatic mediastinitis than other abdominal surgeries, such as pancreaticoduodenectomy, due to the involvement of both the pancreas and the esophageal hiatus; the resulting infection might also be more severe than that caused by cardiac surgery due to the involvement of the intestines, which is likely the origin of Gram-negative bacilli dominant infection.

Several reports have investigated the risk factors for post-operative mediastinitis in cardiothoracic surgery. Although the studies do not agree, obesity [17–19], prolonged surgery time [19, 20], prolonged ICU stay [20], infection at another site [19], and a history of smoking [19] are well-known factors. In our case, both patients were obese and underwent long surgery for more than 5 h. Comparing the two patients, the fatal case was more obese (BMI 27 vs. BMI 25), had a more extended surgery (405 min vs. 296 min), and was diabetic. Mediastinitis should be considered as a complication in patients presenting with causes that could exacerbate an infection or sternum instability, such as obesity, prolonged surgery time, and smoking, including patients with factors that reflect clinical instability, such as prolonged ICU stay.

Mediastinitis manifests within a spectrum that ranges from sub-acute to fulminant and critically ill patients who require immediate intervention in order to prevent death [21]. Although early diagnosis is critical, the condition is difficult to recognize without high clinical awareness of the susceptible populations. Thus, it is extremely difficult to diagnose mediastinitis after abdominal surgery. As the clinical symptoms of mediastinitis cannot be differentiated from other surgical complications, a CT scan helps identify air-fluid levels and pneumomediastinum [2, 9], which are crucial signs for diagnosing mediastinitis.

In treating acute mediastinitis, aggressive early debridement and reconstructive procedures are recommended, but the type of approach remains controversial [1, 9] (Table 2). In cardiac surgery, as the infection is due to sternotomy, reopening the previous sternotomy is the standard procedure [21, 22]. Wound closure is usually delayed, and muscle flaps and vacuum-assisted closure are sometimes used to accomplish closure [1, 21, 22]. In descending necrotizing mediastinitis from odontogenic, pharyngeal, or cervical areas, transcervical or transthoracic approaches are chosen based on the anatomic extent of the inflammation [9]; in severe cases, the two combined approaches are recommended. In reviewing the available literature on enzymatic mediastinitis, chronic pancreatitis was the reported main cause. As such, conservative management was first attempted, but surgical drainage of the mediastinum and abdominal control of the pancreatic leak such as endoscopic retrograde cholangiopancreatography (ERCP) were eventually required [6, 7].

**Table 2:** Type of mediastinitis and recommended treatments

Type of mediastinitis	Treatment	References
Descending mediastinitis	Transcervical or transthoracic debridement Antibiotics	[9]
Direct mediastinitis	Transthoracic debridement Muscle flaps and vacuum-assisted closure Antibiotics	[1, 2, 21]
Enzymatic mediastinitis	Surgical drainage of the mediastinum Abdominal control of the pancreatic leak Antibiotics	[6, 7]

Early debridement and reconstructive procedures are recommended in treating mediastinitis, though the approach is controversial due to the different etiology

Regardless of the type of mediastinitis, the severity of its symptoms and inflammation will determine whether a surgical approach must be chosen, and the main location of the inflammation will determine how the surgery should be performed.

In Case 2, as the disease was due to a transabdominal infection and the inflammation was observed below the bronchial bifurcation, we decided to perform transabdominal drainage. A post-operative CT scan revealed a remarkable reduction in the size of the abscess. Transabdominal drainage has the benefit of determining the infection site, and the drain can also be repositioned in a more effective location. Thus, we suggest transabdominal drainage might be a much simpler yet effective treatment for enzymatic mediastinitis if inflammation is observed in the lower mediastinum.

## Conclusion

We encountered two rare cases of enzymatic mediastinitis caused by total gastrectomy. In both cases, pancreatic leakage was the cause of this severe complication. Although rare, in cases of acute onset of high fever and severe respiratory disorder, the recognition of mediastinitis will enable early diagnosis and intervention. We suggest that transabdominal drainage is an effective approach for treating mediastinitis with inflammation observed below the bronchial bifurcation.

## Data Availability

All data generated during this study are included in this published article.

## Abbreviations

CT: Computed tomography
ERCP: Endoscopic retrograde cholangiopancreatography
BMI: Body mass index
